# Differentiation of industrial hemp strains by their cannabinoid and phenolic compounds using LC × LC-HRMS

**DOI:** 10.1007/s00216-022-03925-8

**Published:** 2022-03-17

**Authors:** Lidia Montero, Sven W. Meckelmann, Hyerin Kim, Juan F. Ayala-Cabrera, Oliver J. Schmitz

**Affiliations:** 1 Applied Analytical Chemistry, University of Duisburg-Essen, Universitaetsstr. 5, 45141 Essen, Germany; 2 Teaching and Research Center for Separation, University of Duisburg-Essen, Universitaetsstr. 5, 45141 Essen, Germany

**Keywords:** Industrial hemp, Cannabinoids, Phenolic compounds, LC × LC–MS, 2D demodulation data treatment

## Abstract

Cannabis is an ancient plant that has been used for therapeutic and recreational purposes. Nowadays, industrial hemp, a variety with low concentration of the psychoactive cannabinoid Δ^9^-tetrahydrocannabinol (THC) and high concentration of non-psychoactive cannabinoids, is getting more and more interest in the food, pharmaceutical, and cosmetic industry. However, cannabis not only contains cannabinoids as bioactive components but also other metabolites like terpenes and phenolic compounds, and the content of these interesting secondary metabolites greatly differs with the genetic variety of the plant. Due to the huge complexity of composition of the cannabis matrix, in this work, a comprehensive two-dimensional liquid chromatography (LC × LC) method has been developed as a very power separation technique coupling a pentafluorophenyl (PFP) and a C18 in the first and second dimensions. Two industrial hemp strains (cookie and gelato) were analyzed to determine the difference in their content of cannabinoids and phenolic compounds. To do this, a new demodulation process was applied for the first time to transform 2D raw data into 1D data which allowed carrying out the chemometric analysis needed to determine the statistical differences between the hemp strains. The cookie strain presented a total of 41 cannabinoid markers, while the gelato strain presented more representative phenolic compounds, in total 24 phenolic compounds were detected as potential markers of this sample. These differences in the chemical composition could determine the industrial destiny of the different hemp strains.

**Graphical abstract:**

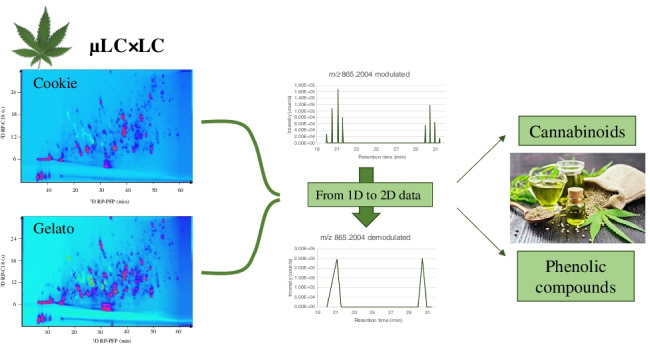

**Supplementary Information:**

The online version contains supplementary material available at 10.1007/s00216-022-03925-8.

## Introduction

Cannabis (*Cannabis sativa* L. and *Cannabis indica* L.) is an herbaceous plant belonging to the *Cannabaceae* widely used for medicinal and recreational purposes by humans. The ancient origin and harvest of cannabis comes from Asia, although it has been commonly cultivated worldwide during the past 5,000 years [1–4]. Traditionally, different parts of the plant have been considered valuable to many different agro-industries, like textile, biofuel, or papermaking industries [5]. Currently, cannabis is well known for its content of cannabinoids. This family of metabolites is formed by a huge variety of terpenophenolic compounds which produce diverse classes of phytocannabinoids [3]. To date, there are more than 144 cannabinoids isolated and identified in cannabis [6, 7].

Cannabinoids are responsible for most of the therapeutic and psychoactive effects of cannabis due to their capability to react and activate receptors present in the endocannabinoid system (cannabinoid receptors) [8]. The two major cannabinoids described in cannabis are Δ^9^-tetrahydrocannabinol (THC) and cannabidiol (CBD). THC is considered for its positive effects on nausea, loss of appetite, or chronic pain treatments. However, it is also mainly responsible for the psychoactive and addictive effects; therefore, the varieties with a high concentration of THC are strictly regulated [9]. On the other hand, CBD is also known for its therapeutical effects, like reducing the effects of anxiety, epilepsy, or cancer, and at the same time, it does not produce any psychoactive effect [10–14].

**Fig. 1:**
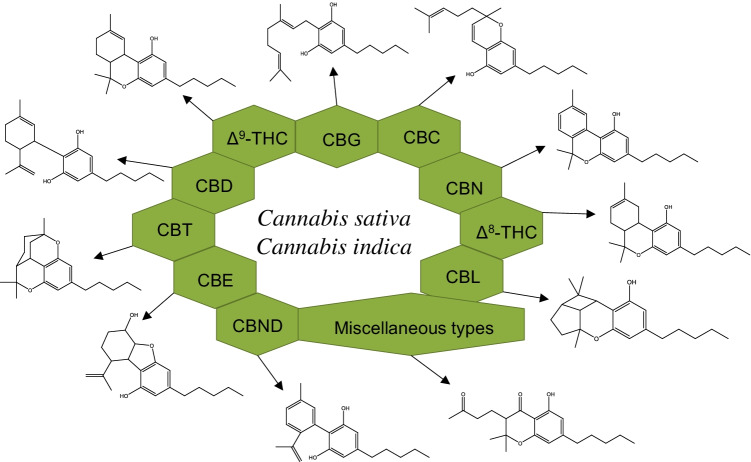
Classification and chemical structure of the eleven more common cannabinoid families present in *C. sativa* and *C. indica*

In general, phytocannabinoids have been classified into eleven groups according to their chemical structure: Δ^9^-THC, CBD, cannabigerol (CBG), cannabichromene (CBC), cannabinol (CBN), Δ^8^-trans-tetrahydrocannabinol (Δ^8^-THC), cannabicyclol (CBL), cannabinodiol (CBND), cannabielsoin (CBE), cannabitriol (CBT), and miscellaneous types [7]. Each of these groups involves their analogs and transformation products [1]. Although cannabinoids can be classified into different subclasses, their chemical structure is similar and based on the CBG-type subclass (Fig. 1).

According to the European Industrial Hemp Association, marihuana contains 1–20% of THC, while industrial hemp does not exceed 0.2% of the psychoactive cannabinoid [2, 4]. Industrial hemp is currently considered as a balanced and complete food with health-promoting effects [2, 15, 16]. Regarding the content of cannabinoids in industrial hemp, these plants present a high concentration of non-psychoactive cannabinoids such as the neutral forms, and the acidic forms like cannabidiolic acid (CBDA) and cannabichromenic acid (CBCA), among many others [2]. Recently, the number of foods, pharmaceutical, and cosmetic products based on industrial hemp that are rich in non-psychoactive cannabinoids has drastically increased [17].

Besides of cannabinoids, industrial hemp contains other important families of secondary plant metabolites like phenolic compounds, terpenes, amides, amines, or phytosterols [4]. All these compounds play an important role together with cannabinoids not only in the organoleptic properties but also in the food and pharmaceutical applications due to their potential bioactive effects. Regarding the phenolic compound content, more than 20 compounds present in cannabis have been detected as flavones, flavonols, and prenylated flavones, which have been related to several health therapeutical activities [4]. Moreover, the presence of phenolic compounds in hemp produces a synergic action over some biological activities related to cannabinoids, producing an enhancement of these interesting bioactivities [18].

However, the chemical composition of hemp cultivars greatly varies depending on multiple factors such as the harvested year, environmental conditions, or the different parts of the plants [2, 15, 19, 20]. One of the most relevant factor that affects the nutritional and second metabolite profile of hemp plants is the genetic diversity between the plants [5, 21, 22]. Thus, each *C. sativa* and *indica* strain presents different chemical compositions and the analysis of the phytochemical profile of the different genetic varieties is important to establish the relationship between composition, medicinal effects, and industrial use [7, 22].

The analysis of hemp extracts is a challenge since there are more than 550 compounds identified in cannabis plants belonging to different chemical families [6] which implies the need for different analytical platforms such as gas chromatography and liquid chromatography coupled to mass spectrometry (GC–MS and LC–MS) for the analysis of all the different components [18, 23]. Besides, NMR [24] and two-dimensional comprehensive gas chromatography (GC × GC) [25] have been used for the analysis of hemp extracts. Liquid chromatography coupled to diode array detection (LC-DAD) and LC–MS methods have been successfully used for the separation and identification of target cannabinoids [7, 22, 23, 26, 27]. Berman et al. developed a method for the identification of 94 cannabinoids, providing the largest LC–MS/MS cannabinoid database. Moreover, LC–MS methods have also been optimized for the analysis of the phenolic fraction of hemp inflorescences [28].

However, there are still some analytical challenges to obtain a complete separation of the whole hemp profile due to the high number of compounds that build the phytochemical composition. For this reason, analytical techniques with high separation power are required to create footprints of the different commercial varieties to establish food authenticity and industrial aims. In this work, a comprehensive two-dimensional liquid chromatography (LC × LC) method together with a new demodulation process that transforms 2D data in 1D data is proposed for the first time for the differentiation of two varieties of industrial hemp according to their cannabinoid and phenolic profile.

## Materials and methods

### Samples and reagents

Two commercial products of dried hemp inflorescences were purchased from a company that produces hemp products. The products (known as cookie and gelato strains) were certified as industrial hemp with a THC content of less than 0.2%. Both of them were indica dominant hybrid (60% *indica*, 40% *sativa*) strains, differentiated by their genetic precedence. While the cookie strain came from the cross of OG Kush with Durban poison strains, the gelato sample was obtained by crossing the Sunset Sherbet with the thin Mint Girl Scout cookie strains.

All solvents used were LC–MS grade. Acetone was purchased from Sigma-Aldrich (Darmstadt, Germany), acetonitrile and methanol were acquired from VWR (Darmstadt, Germany), and formic acid was bought from Fisher Scientific (Schwerte, Germany). Ultrapure water (resistivity 18.2 M Ω cm^−1^) was obtained from a Sartorius Ultrapure Water System (Goettingen, Germany).

### Sample preparation

For the extraction of cannabinoids and phenolic compounds, the dried hemp inflorescences were freeze-dried to remove any remaining water with a vacuum drier (Alpha 1–2 LDplus, Martin Christ, Osterode am Harz, Germany) for 16 h and then mortared into powder. 250 mg of each sample were extracted using 37.5 mL acetone/water 70:30 (*v/v*) as extraction solvent. The mixtures were vortexed for 3 min, sonicated with ultrasonic bath for 30 min, and then centrifuged (Centrifuge 5804R, Eppendorf, Hamburg, Germany) for 10 min (3000 rpm, 5 °C). After the centrifugation, the extract was evaporated under nitrogen stream to remove acetone and then freeze-dried (64 h) to eliminate the water. The powder of both samples was weighed and stored at 4 °C in the darkness until its analysis. Prior to analysis, the extracts were dissolved in water/methanol (50:50, *v/v*).

### µLC × LC-DAD-qTOF MS analysis

The chemical characterization of the hemp extracts was carried out using a two-dimensional liquid chromatography (2DLC) system (Agilent, Walbronn, Germany) coupled with an Agilent 6545 QTOF-MS system (Agilent, Santa Clara, USA). The first dimension (^1^D) was built with a 1260 Infinity HiP micro ALS autosampler module (G1377A), a 1260 Infinity capillary pump (G1376A), a 1260 Infinity column compartment (G1316A), and a 1260 Infinity DAD detector module (G1315C). The second dimension (^2^D) was equipped with a 1290 Infinity II high-speed pump (G7120A) and a 1290 Infinity II DAD detector (G7117B). Moreover, a 1290 Infinity binary pump (G4220A) was used to create a make-up flow rate (additional pump). The coupling of the ^1^D and ^2^D was carried out by an automated controlled 2 ports/4-position dual valve (G1170A) equipped with two 40 µL sampling loops. The operation and control of the system were done using the program OpenLAB ChemStation Edition (Version C.01.07 SR3, Agilent, Santa Clara, USA).

For the ^1^D separation, a Kinetex PFP (150 × 2.1 mm, 1.7 μm, Phenomenex, Torrance, USA) column was used. Ultrapure water with 0.1% (*v/v*) formic acid (solvent A) and methanol with 0.1% (*v/v*) formic acid (solvent B) were used for the gradient elution. The optimized gradient program at a constant flow rate of 0.050 mL min^−1^ was as follows: 5% B, 0 min; 8% B, 5 min; 25% B, 7 min; 35% B, 18 min; 40% B, 19 min; 55% B, 35 min; 65% B, 36 min; 85% B, 52 min; 100% B, 54 min; and 100% B, 65 min. The column temperature was kept at 30 °C while the injection volume was 8 µL. Modulation time was set at 0.5 min. For the ^2^D, a Kinetex C18 (50 × 4.6 mm, 2.6 μm, Phenomenex, Torrance, USA) column was used. Ultrapure water with 0.1% (*v/v*) formic acid and acetonitrile with 0.1% (*v/v*) formic acid were used for solvents A and B, respectively. At the ^2^D, shifted gradient was applied at the following gradient program: 0 min, 5% B; 0.42 min, 10% B; 12 min, 5% B; 12.42 min, 10% B; 33 min, 25% B; 33.42 min, 30% B; 40 min, 25% B; 40.42 min, 45% B; 51 min, 35% B; 51.42 min, 70% B; 60 min, 80% B; and 60.42 min, 100% B. The ^2^D flow rate was set to 2.5 mL min^−1^ and the column temperature was 30 °C. The additional pump for the active modulation was operated with a flow rate of 0.020 mL min^−1^ with ultrapure water containing 0.1% (*v/v*) formic acid. Both ^1^D and ^2^D were connected to two independent DADs. The separation was recorded at 254 nm, saving the wavelength range from 210 to 600 nm. Before entering the MS, the flow was split in a ratio of 7:3 (*v/v*).

The QTOF-MS system worked with an Agilent Dual Jet Stream ion source. The source conditions were as follows: nitrogen gas temperature 325 °C, drying gas 9 L min^−1^, nebulizer 30 psi, sheath gas temperature 300 °C, sheath gas flow 10 L min^−1^, VCap 3500 V, and nozzle voltage 750 V. For the MS qTOF parameters, the fragmentor was set at 380 V while the skimmer and Oct 1 RF Vpp were fixed at 30 V and 750 V, respectively. The samples were analyzed in both ionization modes. The mass range was from *m/z* 100 to 1700. Data-dependent analysis of the top 10 ions was carried out using 20 eV as collision energy. For the operation of the QTOF system and the data acquisition, MassHunter Workstation LC/MS Data Acquisition (Version B.09.00, Agilent, Santa Clara, USA) was used. For the 2D data visualization, LC Image software (Version 2.7r3.1 LC × LC, GC Image, Lincoln, USA) was employed. MassHunter Qualitative Analysis Navigator (Version B.08.00, Agilent, Santa Clara, USA) was used for the MS data analysis.

### Data treatment

For the 2DLC data treatment, firstly, a feature list was created with the software MS-Dial 4.7 (http://prime.psc.riken.jp/compms/msdial/main.html). After that, in order to convert the 2D data in 1D data, the feature list was “demodulated” using a home-made program. This “demodulation” program applies an algorithm that recognized the retention times, *m/z* values, and intensities of the features. Giving the modulation time of the LC × LC analysis, the algorithm combines all the areas of a given *m/z* value that follow a Gaussian distribution along the modulations. Briefly, the program identifies the initial modulation of a peak when it recognizes a *m/z* value at certain intensity and combines all the areas of the following *m/z* values with higher intensity that eluted at exactly the given modulation time. Lastly, it recognizes the last modulation point of the modulated peak when the intensity of that *m/z* value decreases. At the end, the program provides the sum of the areas of the different modulated points for a given feature at the retention time where the maximum intensity was found for each *m/z* value. The program together with the source code and an in-depth explanation will be published separately in the future.

For the statistical analysis, the software Simca 16.0.2 (Sartorius Stedim Data Analytics AB, Umeå, Schweden) was used. Both non-supervised and supervised methods consisting of principle component analysis (PCA) and partial least squares discriminant analysis (PLS-DA) were performed. After that, a suspected targeted analysis of the data was carried out in order to identify cannabinoid-like and phenolic compounds. Finally, in order to distinguish both samples in terms of cannabinoids and phenolic compounds, a cluster was performed.

## Results and discussion

### µLC × LC separation

2DLC provides the separation power that cannot be achieved by conventional 1DLC analysis. The reason for this high separation power is the possibility to analyze a sample by two separation mechanisms that present different selectivity for the analytes. Accordingly, the analytes that are not possible to be separated and coelute in the first column (or first dimension, ^1^D) can be separated in the second column (second dimension, ^2^D). Therefore, 2DLC is the analytical tool of choice for the analysis of very complex samples. In particular, LC × LC is the 2DLC mode preferred to do non-targeted analysis, since the complete sample is separated by both dimensions. In the last years, the application of LC × LC methods for the analysis of complex food and plant samples has greatly increased, showing the expansion on the use of this technique [29]. In this work, a µLC × LC method has been developed for the analysis of the extract of industrial hemp inflorescences [6]. The µLC × LC method was optimized for the separation of cannabinoids and phenolic compounds present in the industrial hemp.

**Fig. 2:**
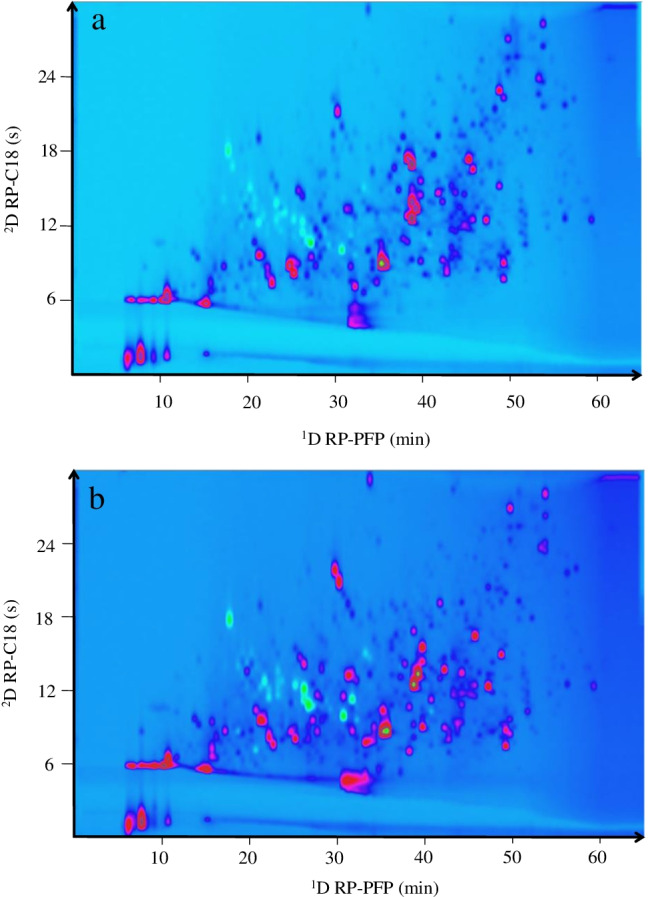
2D plots (254 nm) of the µLC × LC separation achieved coupling a PFP column (^1^D) and a C18 column (^2^D) using a ^2^D multi-segment shifting gradient. **a** Cookie hemp strain; **b** gelato hemp strain

For the ^1^D, a µLC system was used to achieve high reproducible and robust gradients at low flow rates, typically used in ^1^D [30]. Different column combinations were tested for the 2DLC separation of the hemp extract. The combination of HILIC × RP has been successfully used for the separation of very complex phenolic compound and other secondary metabolite mixtures [31–33]; therefore, firstly, hydrophilic interaction liquid chromatography (HILIC) column was tested as ^1^D separation, although it was not possible to achieve an efficient separation to be modulated in the 2D system probably due to the non-polarity of the cannabinoids (data not shown). Then, reversed phase (RP) columns combined with ^2^D HILIC stationary phases (RP × HILIC) like C18 × NH_2_, C18 × HILIC, or C18 × Cys were tested. Using C18 in the ^1^D, a good separation was achieved for the hemp; however, the coupling of this C18 separation in the ^1^D with HILIC produced a high breakthrough and a poor separation of the fractions transferred from the ^1^D into the ^2^D (Figure S1a–c). This effect is due to the high strength mismatch between the mobile phases of RP and HILIC (i.e., the weak solvent in RP is water, which is the strong solvent for HILIC). This fact together with the high sensitivity of HILIC to the injection solvent and the fast analysis carried out in the ^2^D made that the analytes transferred to the ^2^D diluted in the ^1^D solvent were not focused and retained in the ^2^D HILIC column. Finally, a RP × RP combination was checked. The main advantage of the RP × RP coupling is the good mobile phase compatibility between the two separation modes used in both dimensions. For this approach, the ^1^D separation was carried out in a PFP column that presents good properties for the separation of aromatic substances like cannabinoids and phenolic compounds. On the other hand, a short C18 column performed the ^2^D separation. C18 is the most common stationary phase used in the ^2^D [34, 35] due to its beneficial properties to perform fast analysis while keeping a high resolution, and parameters that are required in the ^2^D to carry out the separation of each ^1^D fraction before the next fraction are injected in the ^2^D column. The flow rate used in the ^1^D was optimized at 50 µL/min. Smaller flow rates were not optimal for the ^1^D separation providing a high dead times and therefore long total analysis time as can be observed in Figure S2a–c. Higher flow rates than 50 µL/min were not tested to avoid the collection of large ^1^D fractions that would disrupt the ^2^D separation. Although 50 µL/min is not the optimal flow rate for columns with internal diameter of 2.1 mm, the reproducibility of the ^1^D separation at this flow rate was very precise (Figure S2d). On the other hand, a modulation time of 0.5 min was established in order to achieve a compromise between the undersampling effect and the minimum analysis time required to carry out the ^2^D separation. The combination of two theoretically correlated separation mechanisms could drive to a non-orthogonal 2DLC separation and therefore, the separation of the compounds in the 2D space is limited to the diagonal. In fact, this effect occurred in the present PFP × C18 analysis before the optimization (Figure S1d), where the same ^2^D gradient was used during the whole 2DLC analysis (^2^D full gradient). To improve the orthogonality, different mobile phases were tested in both dimensions (methanol was used as organic solvent in PFP, and acetonitrile was used for the separation in the C18 ^2^D). Besides, the ^2^D gradient was tailored according to the ^1^D eluted fractions along the whole 2DLC analysis developing a ^2^D multi-segment shifting gradient. The highly improved orthogonality obtained for the separation of the cookie and gelato hemps after the optimization of the tailored ^2^D gradient can be observed in Fig. 2. To qualify the gain in the separation after the optimization and to quantify the effect of the ^2^D multi-segment shifting gradient, the peak capacity and the orthogonality of both 2DLC methods, the ^2^D full gradient (Figure S1d), and the ^2^D shifting gradient (Fig. 2) were calculated. The practical peak capacity (^2D^n_*c,practical*_) was calculated according to Li *et al.* [36], the orthogonality was estimated following the asterisk equations (A_O_) [37], and the corrected peak capacity (^2D^n_*c,corr*._) was calculated multiplying the ^2D^n_*c,practical*_ and the A_O_. For the ^1^D, a peak width media of 1 and 0.8 min was calculated for the full and shifting gradient, respectively, while the peak width media of the ^2^D was 3.3 and 0.4 s for the two respective settings. The peak capacities of the ^1^D (^1^n_*c*_) for the full gradient and the shifting gradient methods were 59 and 75, respectively. On the other hand, the peak capacities of the ^2^D (^2^n_*c*_) in the full gradient method was 10 and for the shifting gradient method was 62. Therefore, the ^2D^n_*c,practical*_ values were 442 and 3080 for the ^2^D full gradient and the ^2^D shifting gradient, respectively. Regarding the orthogonality, a gain of 23% was obtained after the optimization of the ^2^D gradient (A_O_ = 40% for ^2^D full gradient and A_O_ = 63% for the ^2^D shifting gradient). To have a more realistic value about the peak capacity, the ^2D^n_*c,practical*_ was corrected by the real 2D space occupied by the separated compounds (A_O_). The ^2D^n_*c,corr*._ values for the full gradient and shifting gradient were 170 and 1940, respectively. Although, as mentioned above, the peak capacity value should not be considered as a real number of separated peaks, this value is very useful not only to compare the improvement between different 2DLC methods carried out with the same setup during the optimization process but also to confirm the separation gain that 2DLC offers in comparison to conventional 1D separation for this kind of complex samples. In this work, the peak capacity achieved by 2DLC is much higher than the individual peak capacity obtained by the corresponding 1D alone. However, it is also important to remark that the 2DLC method is always accompanied by a big optimization and development effort and it involves specific instrumentation as well as a difficult data treatment as will be discussed in the following section. For this reason, the application of a 2DLC method should be always justified by a great gain in peak capacity.

### Data treatment in µLC × LC

Although 2DLC has been established as a suitable alternative to conventional one-dimensional systems for the separation of very complex matrices in the last years, there is still a limitation on its use related to the data treatment. 2D raw data presents a matrix where each compound or feature is modulated several times due to the fractionation of each ^1^D peak. Therefore, the signal of a single compound is divided with a time frame corresponding to the modulation time in the raw data. The modulation of the signal makes it not possible to apply automatic data treatment to create a feature list that allows the typical workflow for the identification and statistical analysis. Different efforts have been done to solve this limitation, for example, by the compression of the data applying a selection of the region of interest (ROI) in the MS dimension, a one-dimensional wavelet analysis of the previous *m/z* ROI values, and finally a time windowing strategy of the compressed data [38], or by the development of a new 2DLC concept called LC + LC, where the modulation time is increased to avoid the modulation of the peaks, that way, each peak is analyzed only once by the ^1^D and the ^2^D, achieving only one signal per compound [39]. However, the reported solutions present some limitations like the complexity of the data treatment workflow or the loss of ^1^D resolution due to undersampling effects. In this work, a new data treatment has been used for the first time to convert 2D data feature analysis into a 1D data file that only shows one intensity or area per detected feature. This so-called demodulation process allows the use of common data analysis strategies (e.g., PCA) for the comparison between the chemical compositions of the cookie and gelato hemp samples acquired by a comprehensive 2D µLC × LC-HRMS analysis.

The analyses of the gelato and cookie samples were done in triplicate. Then, MS-Dial software was used for the identification and the alignment of all the features detected in the µLC × LC-HRMS analysis of the cookie and gelato hemp strains. The result of this alignment provided 75,387 features which included the divided signals of the modulated analytes. This feature list was introduced in the new demodulation tool. Although the signal of one compound is divided in modulation time frames, it follows a typical 1D Gaussian trend peak. The demodulation tool was programed to recognize the Gaussian trend of one *m/z* value that appears with time intervals equal to the modulation time and to combine the areas of all the modulated signals in the feature that corresponded to the maximum peak height. That way, the 2D modulated data are now reconstructed into 1D peaks that combine the total area of the 2D modulated peaks, that is, the demodulation tool transforms 2D data in 1D data. A graphical example of the demodulation process can be observed in Figure S3, where the ion *m/z* 865.2004 was modulated four times in the total retention time of 20.05, 20.55, 21.15, and 21.61 min and four times more at 29.99, 30.47, 30.95, and 31.44 min. After the demodulation, it can be seen how the areas of the corresponding modulations were summed up in the retention time of the maximum peak height. This tool provides a huge advantage in the global 2DLC methodology, since up to now, some omic applications have limited the use of this high resolution and separation technique due to the lack of powerful program able to deal with the complex 2DLC data treatment.

After applying the demodulation process, the feature list was reduced to 37,961 features. However, this number was still too high and therefore, filters were applied to obtain high quality features. Firstly, features with an intensity lower than 1 × 10^5^ counts were discarded. Then, features that presented a relative standard deviation (RSD) higher than 50% across the triplicates were eliminated. This threshold was chosen as a compromise between analytical error and 2DLC repeatability (affected by both dimensions), since slight differences in the ^1^D retention time can have high effect in both the ^2^D retention time and area. Finally, all the features with a signal-to-noise ratio lower than 3 were also discarded of the data matrix. After this filtering, 5,296 features were considered for the statistical analysis.

Chemometric analysis was applied to evaluate the statistical differentiation of the cookie and gelato samples. First, a non-supervised method such as PCA was applied (Figure S4a) and after that, a PLS-DA was performed as a supervised method to describe the model (Figure S4b). In the loading plots of both statistical analyses, it is possible to observe a substantial number of features accumulated in the edge of the plot, which could be responsible for the statistical differentiation of the samples. Moreover, in the PCA and PLS-DA score plots, the PC1 was able to explain 65.2% of the samples’ variance. These results indicate that the two hemp samples presented a different chemical composition that could be used for authentication purposes as well as for the application in specific therapeutical, pharmaceutical, or cosmetic motivations depending on the composition of those hemp strains.

### Differentiation of the cannabinoids and phenolic compounds in the cookie and gelato strains

The composition of industrial hemp is highly affected by the genetic variation of the plant, and consequently, the composition of bioactive compounds is different in each of the strains [4, 22]. Thus, the variety in the chemical composition and, particularly, in bioactive compounds can increase the applications of each hemp strain to very specific functions in the industry. The interest of this work was to evaluate the composition of cannabinoids and phenolic compounds of two commercially available industrial hemps and to assess possible differences between them. To do that, a flagging approach was carried out in the whole demodulated and filtered data.

For the suspected detection of cannabinoids, the database provided in the work of Berman *et al. *[7] was used to search for the most studied cannabinoids using the corresponding *m/z* values of [M-H]^−^ ions as well as their typical MS/MS product ions. Besides, the most common product ions were selected to find other possible cannabinoids in the hemp strains.

Most of the cannabinoids found could be grouped into two families. On one hand, cannabinoids presented the typical fragment ion at *m/z* 179.1078 corresponding to the pentylresorcinol structure (Figure S5a). The second family of cannabinoid compounds was found to have a product ion at *m/z* 195.1031 corresponding to a hydroxylated pentylresorcinol (Figure S4b). For this last type of cannabinoids, only few information was available. In fact, only Berman *et al.* have reported them although they only classified and identified them as additional phytocannabinoids and provided the molecular formula. In particular, they found 27 compounds that were tentatively identified as potential phytocannabinoids by accurate mass and fragmentation patterns related to the already identified phytocannabinoids. Four of these 27 compounds were phytocannabinoid isomers at *m/z* 329.2122 and 373.2021 that presented the *m/z* 195.1027 as one of the main fragments [7]. Therefore, this fragment ion was also considered for the flagging approach to tentatively identify other potential phytocannabinoids. Besides these two families of cannabinoids, other cannabinoids described in the literature were considered.

**Fig. 3:**
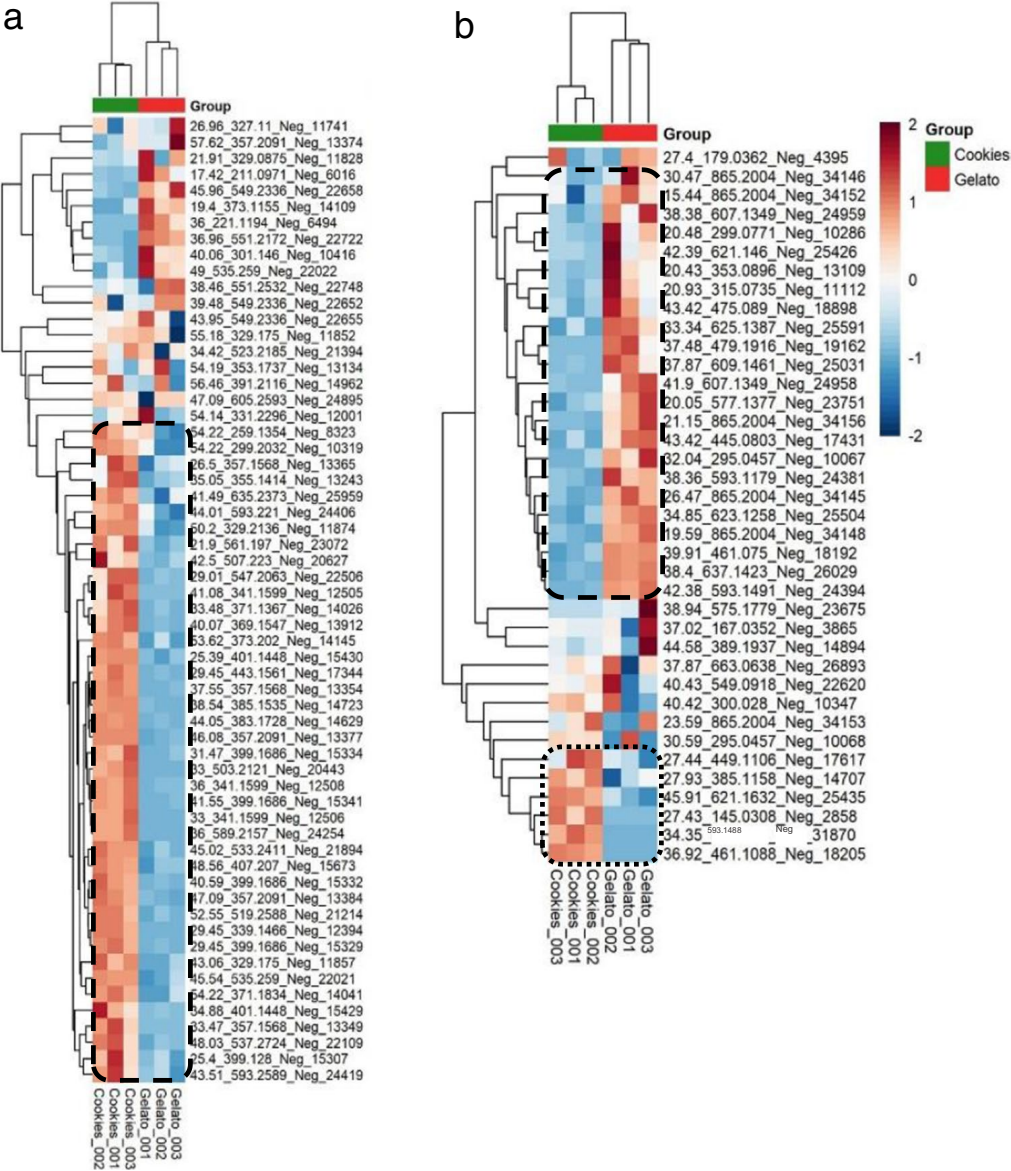
Heat maps of suspected cannabinoids (**a**) and phenolic compounds (**b**)

After the flagging approach was done, the selected features were submitted to statistical analysis to determine the pattern of cannabinoids between the cookie and gelato sample. The heat map of the cannabinoids present in both samples is shown in Fig. 3a. As can be observed, it was possible to distinguish the two samples by their content on cannabinoids. Besides, this heat map revealed a cluster group of closely related compounds (marked in dash line) that were characteristic of the cookie sample. Moreover, it was also possible to appreciate that the cookie sample was richer in cannabinoids, being considered as potential markers since they presented a higher trend in this strain. The identification of these potential markers is shown in Table 1. Among them, there were some cannabinoids previously reported. For instance, the peaks observed at 46.08 and 47.07 min with an *m/z* value of 357.2091 and a fragmentation pattern consisted of *m/z* 313.2189, 245.1545, and 191.1080 could be assigned to the acidic forms CBDA, CBCA, CBLA, or CBRA. All these compounds as well as the acidic form of THC, the THCA, are isomers. However, THCA was not considered as possible identification due to the low concentration of THC in the industrial hemp samples. CBDA has been shown as the major compound in industrial hemp, and it is usually more abundant than the corresponding neutral form CBD. This fact is explained by the biosynthesis of these compounds since the phytocannabinoids are synthesized in the plant as acids [6, 22, 40]. The extracted ion chromatogram (EIC) of the ion *m/z* 357.2091 presented the maximum intensity at 46.08 min, so the compound eluted at that retention time could be tentatively identified as CBDA. Other identified cannabinoids belonged to the varinic acid-C3 type like CBDVA, CBCVA, or CBLVA (*m/z* 329.175 corresponding to [M-H]^−^ ion and its product ions *m/z* 285.1878, 217.1203, and 163.0774), as well as the neutral-C4 form type cannabinoids identified as CBD-C4, CBC-C4, or CBL-C4 (*m/z* 299.2032 assigned to [M-H]^−^ ion and its product ions *m/z* 269.1554, 231.1385, and 213.0899), according to the classification done in the database reported by Berman *et al. *[7]. Moreover, additional phytocannabinoids described in the database without the complete characterization or name were also identified by means of the deprotonated molecule as important compounds for the characterization of the cookie sample. Following the nomenclature provided in the database, these compounds were named as the isomers 11a, 11b, 11c, or 11d (*m/z* 329.2136); 12a, 12b, 12c, or 12d (*m/z* 373.2020); 13c (*m/z* 327.1975); and 14a or 14b (*m/z* 371.1835).

**Table 1:** Differentiated cannabinoids found in the cookie hemp strain

	Peak ID	Feature ID (RT_MZ_Pol_id)	Total Rt (min)	[M-H]^−^	MS/MS	Formula	Identification	Theoretical [M-H]^−^	ppm
Reported cannabinoids	1c	46.08_357.2091_Neg_13377	46.08	357.2091	313, 245, 191	C22H29O4	CBCA/CBDA/CBLA/CBRA	357.2066	7.0
	2c	47.09_357.2091_Neg_13384	47.09	357.2091	313, 245, 192	C22H29O4	CBCA/CBDA/CBLA/CBRA	357.2066	7.0
	3c	50.2_329.2136_Neg_11874	50.20	329.2136	261, 195, 107	C21H29O3	11a/11b/11c/11d*	329.2116	6.1
	4c	53.62_373.202_Neg_14145	53.62	373.2020	355, 329, 261, 195, 179	C22H29O5	12a/12b/12c/12d*	373.2015	1.3
	5c	54.22_259.1354_Neg_8323	54.22	327.1975	259, 231, 174	C21H27O3	13c*	327.1960	4.6
	6c	54.22_371.1834_Neg_14041	54.22	371.1834	327, 285, 259	C22H27O5	14a/14b*	371.1859	− 6.7
	7c	43.06_329.175_Neg_11857	43.06	329.1750	285, 217, 163	C20H25O4	CBDVA/CBCVA/CBLVA	329.1752	− 0.6
	8c	54.22_299.2032_Neg_10319	54.22	299.2032	269, 231, 213, 174, 142	C20H27O2	CBD-C4/CBC-C4/CBL-C4	299.2011	7.0
Phytocannabinoids with *m/z* 179 as main product ion	9c	21.9_561.197_Neg_23072	21.90	561.1779	532, 219, 193, 175, 123	C31H29O10		561.1766	2.3
	10c	29.01_547.2063_Neg_22506	29.01	547.2063	385, 179	C24H35O14		547.2032	5.7
	11c	33_341.1599_Neg_12506	33.00	341.1599	179	C17H25O7		341.1606	− 2.1
	12c	33_503.2121_Neg_20443	33.00	503.2121	179	C23H35O12		503.2134	− 2.6
	13c	36_341.1599_Neg_12508	36.00	341.1599	179	C17H25O7		341.1606	− 2.1
	14c	36_589.2157_Neg_24254	36.00	589.2117	179	C33H33O10		589.2079	6.4
	15c	38.54_385.1535_Neg_14723	38.54	385.1504	179	C18H25O09		385.1504	0.0
	16c	41.08_341.1599_Neg_12505	41.08	341.1599	179	C17H25O7		341.1606	− 2.1
	17c	44.05_383.1728_Neg_14629	44.05	383.1728	179	C19H27O8		383.1711	4.4
	18c	45.54_535.259_Neg_22021	45.54	535.2576	329, 311, 179	C28H39O10		535.2549	5.0
	19c	52.55_519.2588_Neg_21214	52.55	519.2588	451, 313, 245, 179	C28H39O9		519.2600	− 2.3
	20c	26.5_357.1568_Neg_13365	26.50	357.1568	195, 123	C17H25O8		357.1555	3.6
Phytocannabinoids with *m/z* 195 as main product ion	21c	25.39_401.1448_Neg_15430	25.39	401.1448	399, 195	C18H25O10		401.1453	− 1.2
	22c	25.4_399.128_Neg_15307	25.40	399.1280	195	C18H23O10		399.1297	− 4.3
	23c	29.45_339.1466_Neg_12394	29.45	339.1466	195	C17H23O7		339.1449	5.0
	24c	29.45_399.1686_Neg_15329	29.45	399.1686	195	C19H27O9		399.1661	6.3
	25c	29.45_443.1561_Neg_17344	29.45	443.1561	195	C20H27O11		443.1559	0.5
	26c	31.47_399.1686_Neg_15334	31.47	399.1686	195, 123	C19H27O9		399.1661	6.3
	27c	33.47_357.1568_Neg_13349	33.47	357.1568	195	C17H25O8		357.1555	3.6
	28c	34.88_401.1448_Neg_15429	34.88	401.1448	383, 357, 339, 275, 195, 177	C18H25O10		401.1453	− 1.2
	29c	37.55_357.1568_Neg_13354	37.55	357.1568	195	C17H26O8		357.1555	3.6
	30c	40.59_399.1686_Neg_15332	40.59	399.1686	195	C19H27O9		399.1661	6.3
	31c	41.55_399.1686_Neg_15341	41.55	399.1686	195	C19H27O9		399.1661	6.3
Other phytocannabinoids	32c	43.51_593.2589_Neg_24419	43.51	593.2589	575, 557, 489, 425, 393, 371, 327, 301, 195	C30H41O12		593.2604	− 2.5
	33c	33.48_371.1367_Neg_14026	33.48	371.1367	191, 165	C17H23O9		371.1348	5.1
	34c	35.05_355.1414_Neg_13243	35.05	355.1414	194, 177, 151	C17H23O8		355.1398	4.5
	35c	40.07_369.1547_Neg_13912	40.07	369.1547	271, 165	C18H25O8		369.1555	− 2.2
	36c	41.49_635.2373_Neg_25959	41.49	635.2373	591, 387, 369, 343, 325, 193, 173	C31H40O14		635.2345	4.4
	37c	42.5_507.223_Neg_20627	42.50	507.2230	345, 301, 283, 232, 173	C26H35O10		507.2236	− 1.2
	38c	44.01_593.221_Neg_24406	44.01	593.2210	575, 549, 389, 327, 309, 259, 191, 173	C29H37013		593.2240	− 5.1
	39c	45.02_533.2411_Neg_21894	45.02	533.2411	193	C28H37O10		533.2392	3.6
	40c	48.03_537.2724_Neg_22109	48.03	537.2724	375, 357, 331, 245, 191	C28H41O10		537.2705	3.5
	41c	48.56_407.207_Neg_15673	48.56	407.2070	193	C22H31O07		407.2075	− 1.2

^*^Classification by Berman *et al.*

The peak ID refers to the peak labeling in Figure S7

The rest of the selected compounds for the cookie sample were not previously identified. As mentioned above, these potential unknown cannabinoids could be divided in two groups according to the main product ion shown in their fragmentation pathway. In total, eight additional phytocannabinoids presented as main in-source fragment ion *m/z* 179.1078 and fourteen phytocannabinoids showed the *m/z* 195.1031 as major fragment in the outstanding compounds in cookie hemp. The retention time, accurate mass, molecular formula, and the MS/HRMS fragments of all of them are summarized in Table 2. A possible molecular structure of one of each phytocannabinoid group is proposed in Figure S5c–d.

**Table 2:** Differentiated phenolic compounds found in the gelato hemp strain

Peak ID	Feature ID (RT_MZ_Pol_id)	Total Rt (min)	[M-H]^−^	MS/MS	Formula	Identification	Theoretical [M-H]^−^	ppm	Phenolic classification
1 g	15.44_865.2004_Neg_34152	15.44	865.2004	739, 713, 696, 575, 451, 287, 125	C45H37O18	Procyanidin trimer	865.1980	2.8	Procyanidin
2 g	19.59_865.2004_Neg_34148	19.59	865.2004	739, 713, 577, 451, 287, 125	C45H37O18	Procyanidin trimer	865.1980	2.8	Procyanidin
3 g	20.05_577.1377_Neg_23751	20.05	577.1377	425, 407, 289	C30H25O12	Procyanidin dimer	577.1346	5.4	Procyanidin
4 g	20.43_353.0896_Neg_13109	20.43	353.0896	191, 179, 135	C16H17O9	Chlorogenic acid	353.0878	5.1	Phenolic acid
5 g	20.48_299.0771_Neg_10286	20.48	299.0771	137, 93	C13H15O8	Salicylic acid-hexoside	299.0767	1.3	Phenolic acid
6 g	20.93_315.0735_Neg_11112	20.93	315.0735	153, 109	C13H15O9	Dihydroxybenzoic acid-hexoside // Protocatechuic acid-hexoside	315.0716	6.0	Phenolic acid
7 g	21.15_865.2004_Neg_34156	21.15	865.2004	739, 713, 577, 425, 289, 125	C45H37O18	Procyanidin trimer	865.1980	2.8	Procyanidin
8 g	26.47_865.2004_Neg_34145	26.47	865.2004	739, 575, 425, 245, 125	C45H37O18	Procyanidin trimer	865.1980	2.8	Procyanidin
9 g	27.4_179.0362_Neg_4395	27.40	179.0362	161, 151, 135, 107, 59	C9H7O4	Caffeic acid	179.0344	10.1	Phenolic acid
10 g	30.47_865.2004_Neg_34146	30.47	865.2004	847, 739, 713, 575, 449, 369, 287, 125	C45H37O18	Procyanidin trimer	865.1980	2.8	Procyanidin
11 g	32.04_295.0457_Neg_10067	32.04	295.0457	251, 226, 154, 129, 111, 85	C13H11O8	NI	295.0459	− 0.7	
12 g	33.34_625.1387_Neg_25591	33.34	625.1387	463, 301, 178	C27H29O17	Quercetin-dihexoside	625.1405	− 2.9	Flavonol
13 g	34.85_623.1258_Neg_25504	34.85	623.1258	285	C27H27O17	Luteolin-dihexoside // kaempferol-dihexoside	623.1248	1.6	Flavone
14 g	37.48_479.1916_Neg_19162	37.48	479.1916	316, 301, 285	C24H31O10	NI	479.1923	− 1.5	
25 g	37.87_609.1461_Neg_25031	37.87	609.1461	300	C27H29O16	Quercetin rutinoside	609.1456	0.8	Flavonol
16 g	38.36_593.1179_Neg_24381	38.36	593.1179	285	C26H25O16	Luteolin hexuronide-pentoside // kaempferol hexuronide-pentoside	593.1143	6.1	
17 g	38.38_607.1349_Neg_24959	38.38	607.1349	563, 319, 299, 284, 161, 85	C28H31O15	Hispidulin hexoside-penturonide // diosmetin hexoside-penturonide // chrysoeriol hexoside-penturonide // rhamnocitrin hexoside-penturonide	607.1299	8.2	Flavone
18 g	38.4_637.1423_Neg_26029	38.40	637.1423	299, 284	C28H29O17	Hispidulin hexoside-hexuronide // diosmetin hexoside-hexuronide // chrysoeriol hexoside-hexuronide // rhamnocitrin hexoside-hexuronide	637.1405	2.8	Flavone
19 g	39.91_461.075_Neg_18192	39.91	461.0750	285	C21H17O12	Luteolin hexuronide // kaempferol hexuronide	461.0720	6.5	Flavone
20 g	41.9_607.1349_Neg_24958	41.90	607.1349	563, 319, 299, 284, 161, 85	C28H31O15	Hispidulin hexoside-penturonide // diosmetin hexoside-penturonide // chrysoeriol hexoside-penturonide // rhamnocitrin hexoside-penturonide	607.1299	8.2	Flavone
21 g	42.38_593.1491_Neg_24394	42.38	593.1514	299, 284, 269	C27H29O15	Hispidulin hexoside-pentoside // diosmetin hexoside-pentoside // chrysoeriol hexoside-pentoside // rhamnocitrin hexoside-pentoside	593.1509	0.8	Flavone
22 g	42.39_621.146_Neg_25426	42.39	621.1460	299, 284	C28H29O16	Hispidulin deoxyhexoside-hexuronide // diosmetin deoxyhexoside-hexuronide // chrysoeriol deoxyhexoside-hexuronide // rhamnocitrin deoxyhexoside-hexuronide	621.1456	0.6	Flavone
23 g	43.42_445.0803_Neg_17431	43.42	445.0803	269	C21H17O11	Genistein hexuronide	445.0771	7.2	Isoflavone
24 g	43.42_475.089_Neg_18898	43.44	475.0890	299, 284, 227	C22H20O12	Hispidulin hexuronide // diosmetin hexuronide // chrysoeriol hexuronide // rhamnocitrin	475.0877	2.7	Flavone

*NI*, no identified

The peak ID refers to the peak labeling in Figure S7

On the other hand, a similar strategy was followed for the suspected analysis of phenolic compounds. In this case, Phenol-Explorer was used as database for the search of phenolic compounds in both samples [41]. This search was done by monitoring the aglycone ions which usually are the main fragments of phenolic compounds. The heat map of the phenolic compounds found in the samples is depicted in Fig. 3b. As it happened with the cannabinoid content, it was also possible to differentiate the samples according to their phenolic compound content. Interestingly, in this case, gelato sample showed a group of closely related compounds highlighted in comparison to the gelato strain (dashed line). Thereby, these compounds were considered as potential authentication markers for the gelato sample.

The potential phenolic markers of the gelato sample are listed in Table 2. These compounds belonged to different classes of phenolic compounds. One of the main phenolic compound classes was the procyanidins, which are polymeric phenolic compounds formed by the link of catechin or epicatechin units and constitute the second most abundant group of phenolic compounds in nature. They are responsible not only for some organoleptic properties like astringency but also several therapeutical properties have been related to them, like antioxidant, anticancer, cardioprotective, antimicrobial, antiviral, neuro-protective, and anti-inflammatory activities, among others [42, 43]. The presence of this phenolic class in *C. sativa* has been recently reported for the first time [23]. In that work, two procyanidin dimers and two trimers were identified. In this work, one procyanidin dimer and five different trimers were shown as characteristic compounds from the gelato strain. For example, the procyanidin trimers were tentatively identified thanks to the accurate mass of the precursor [M-H]^−^ ion at *m/z* 865.2004 and to the typical fragmentation pattern of procyanidins consisting of the neutral losses of one or two units of (epi)cathechin leading to the precursor ions at *m/z* 577.1281 or 575.1200 and 289.0728 or 287.0557, respectively, depending on where these losses were produced (terminal or the intermediate units). Besides, typical fragment ions from the Retro-Dial-Alder reaction were observed like *m/z* 739.1757, 713.1755, 451.1026, 425.0867, and 125.0254 (Figure S6). Another representative family of phenolic compounds described as markers of the gelato strain was the flavone class. Several compounds presented common fragment ions at *m/z* 299.0556 and 284.0320. The ion *m/z* 299.0556 was identified as the aglycone ion ([C_16_H_11_O_6_]^−^, − 1.7 ppm) while the ion *m/z* 284.0320 resulted from the loss of a methyl group from the aglycone ([C_15_H_8_O_6_]^−•^, − 2.2 ppm). The molecular formula C_16_H_11_O_6_ has been related to different aglycones such as the methylflavones hispidulin, diosmetin, chrysoeriol, and rhamnocitrin. The compounds identified with these fragment ions presented different glycosidic pattern from the aglycone (*m/z* 299.0556). For example, it was possible to detect the presence of a hexoside-penturonide glycosylation (*m/z* 607.1349) as well as hexoside-hexuronide (*m/z* 637.1423), hexoside-pentoside (*m/z* 593.1514), hexuronide (*m/z* 475.0890), and deoxyhexose-hesuronide (*m/z* 621.1460) glycosidic moieties attached to the aglycone. To the best of our knowledge, there is no previous work reporting the presence of hispidulin or rhamnocitrin derivatives in cannabis, although diosmetin and chrysoeriol have already been detected in this plant [4, 23]. Therefore, these methylflavone derivates could be tentatively identified as diosmetin- or chrysoeriol-related compounds. However, more investigation should be done to confirm the identity of these compounds although it can be concluded that all these compounds are derived from the same aglycone (*m/z* 299.0556) and they have a defined glycosidic pattern.

The rest of the detected compounds belonged to the flavonol, flavone, isoflavone, and phenolic acid classes. Among the flavonols, quercetin-dihexoside and quercetin-rutinoside were identified by their [M-H]^−^ ions (*m/z* 625.1387 and 609.1461, respectively) and the common fragment ion *m/z* 301.0352 ([C_15_H_9_O_7_]^−^, − 0.6 ppm) corresponding to the quercetin aglycone. Three flavones were also highlighted in the gelato strain. In this case, the found aglycone fragment ion presented an *m/z* value of 285.0416 ([C_15_H_9_O_6_]^−^, 4.0 ppm), which corresponds to the isomers luteolin or kaempferol. The three derivatives of these aglycones were luteolin or kaempferol dihexoside (*m/z* 623.1258), luteolin or kaempferol hexuronide (*m/z* 461.0750), and luteolin or kaempferol hexuronide-penstoside (*m/z* 593.1179). Quercetin, luteolin, and kaempferol derivates have been extensively described in *C. sativa* varieties [4, 28]. The isoflavone detected in the group of highlighted compounds in gelato was tentatively identified as genistein hexuronide with a [M-H]^−^ ion of *m/z* 445.0803 and the main fragment ion of *m/z* 269.0460 ([C_15_H_9_O_5_]^−^, 1.7 ppm).

Finally, phenolic acids also form part of the phenolic profile of cannabis. Caffeic acid, cinnamic acid, benzoic acid, coumaric acid, and ferulic acid are some of the phenolic acids that have been identified in different cannabis plants [4, 28, 44]. Here, several phenolic acids were identified in this group like caffeic acid (*m/z* 179.0362), salicylic acid-hexoside (*m/z* 299.0771), and dihydroxybenzoic acid-hexoside or protocatechuic acid-hexoside (*m/z* 315.0735).

Therefore, the chemical composition of the cookie strain presented a richer cannabinoid profile in comparison with the gelato strain, showing several cannabinoids that distinguished that sample. In contrast, the gelato strain was more related to the content on phenolic compounds, being some of them characteristic analytes of this sample.

The peak labeling of all the tentatively identified compounds in cookie and gelato samples can be observed in Figure S7.

## Conclusions

In this work, a µLC × LC-HRMS method is used for the analysis of two commercial industrial hemp strains (i.e., cookie and gelato). Two reversed phase modes were coupled in the ^1^D and ^2^D. To increase the orthogonality of these correlated modes, a ^2^D multi-segment shifting gradient was optimized that enhanced the orthogonality in 23% and produced a peak capacity eleven times higher. After the analysis, a demodulation process, which is able to transform 2D data into 1D data, was applied for the first time. With this process, the 2D data treatment of a very complex sample (75,387 features) was easily handled for the statistical and identification process. Due to the high variability in bioactive compounds between hemp varieties and strains, a suspected analysis of the phytocannabinoids and phenolic compounds present in both samples was carried out. Cookie sample presented a higher content and a higher number of characteristic cannabinoids that could be considered as potential markers of this strain. Among them, the acidic form of CBD (CBDA) or its isomer, varinic acid-C3 type, and neutral-C4 form type phytocannabinoids were identified together with several cannabinoids that presented a common MS/MS pathway not previously reported. On the other hand, the gelato sample was richer in phenolic compounds, among which an important number of potential markers were highlighted. Procyanidins and diosmetin or chrysoeriol glycosidic derivates were the major compounds that were characteristic of this sample. Therefore, this study reveals the different bioactive compound profiles between two industrial hemp varieties. The chemical characterization of them would be of great interest for pharmaceutical, food, or cosmetic applications that could be targeted to specific interests according to the properties offered by the compounds present in each strain. Another conclusion of this work is that the number of secondary metabolites in cannabis is exponentially growing more and more, and further studies are needed for achieving a high confident identification level of all the bioactive compounds responsible for all the interesting applications of cannabis.

## Supplementary Information

Below is the link to the electronic supplementary material.

Supplementary file1 (DOCX 4977 KB)
