# Supplementary material for: Differentiation of industrial hemp strains by their cannabinoid and phenolic compounds using LC × LC-HRMS
Source: Anal Bioanal Chem. 2022 Mar 17;414(18):5445–59. doi: 10.1007/s00216-022-03925-8 (PMC9242925; doi:10.1007/s00216-022-03925-8)
Supplement: Supplementary file 1 — Supplementary file1 (DOCX 4977 KB) [file 216_2022_3925_MOESM1_ESM.docx]

**Supplementary Information**

**Differentiation of industrial hemp strains by their cannabinoid and phenolic compounds using LC×LC-HRMS**

Lidia Montero^a,b,*^, Sven W. Meckelmann^a,b^, Hyerin Kim^a,b^, Juan F. Ayala-Cabrera^a,b^, Oliver J. Schmitz^a,b^

^(a)^ Applied Analytical Chemistry, University of Duisburg-Essen, Universitaetsstr. 5, 45141 Essen, Germany

^(b)^ Teaching and Research Center for Separation, University of Duisburg-Essen, Universitaetsstr. 5, 45141 Essen, Germany

**^*^**Corresponding author: Lidia Montero

Applied Analytical Chemistry,

University of Duisburg-Essen

Universitatsstr. 5, D‒45141, Essen, Germany

Phone: +49 (0) 201 183 4599

E-mail: [lidia.montero@uni-due.de](mailto:lidia.montero@uni-due.de)

**Fig S1** 2D plots (254 nm) of different LCxLC combinations used during the optimization of the µLCxLC method. a) C18xNH2; b) C18xHILIC; c) C18xCys; and d) PFPxC18 using 2D full gradient

**Fig S2** Optimization of the 1D flow rate in the PFP column: a) 10 µL/min; b) 20 µL/min; c) 50 µL/min; d) reproducibility of three analysis done in the PFP column at 50 µL/min.

**Fig S3** Raw data, process data and graphical representation of the demodulation process used in this work to transform 2D data into 1D data. a) 2D raw data before the demodulation of the ion *m/z* 865.2004; b) 2D raw data after the demodulation of the ion *m/z* 865.2004; c) graphical representation of the data before the demodulation process; d) representation of the data after the demodulation process of the ion *m/z* 865.2004

**Fig S4** Loading and score plots of the PCA (a) and PLS (b) analysis of the cookie and gelato samples

**Fig S5** Main fragments found in the highlighted cannabinoids in the cookie sample. a) CBDA structure and MS/MS fragmentation pathway which gives rise to the pentylresorcinolion as characteristic fragment (*m/z* 179.1078); b) proposed hydroxylated pentylresorcinol (*m/z* 195.1031); c) proposed chemical structure and MS/MS fragmentation pathway of the ion *m/z* 383.1728 detected at 44.05 min and presented the fragment ion m/z 179.1078 as main fragment (feature ID: 44.05_383.1728_Neg_14629_Neg_12394); d) proposed chemical structure and MS/MS fragmentation pathway of the ion *m/z* 339.1466 detected at 29.45 min and presented the fragment ion *m/z* 195.1027 as main fragment (feature ID: 29.45_339.1466_Neg_12394)

**Fig S6** MS/MS pathway and spectra of one of the procyanidin trimers found in the gelato sample (*m/z* 865.1980)

**Fig S7** Peak labeling of the tentatively identified compounds present in Table 1 for the samples cookie (a) and gelato (b).

**Fig S1**

**Fig S2**

**Fig S3**

**Fig S4**

**Fig S5**

**Fig S6**

**Fig S7**
